# Role of Cardiovascular Magnetic Resonance in Cardiac Amyloidosis: A Narrative Review

**DOI:** 10.3390/jpm14040407

**Published:** 2024-04-11

**Authors:** Nicola Maggialetti, Andrea Torrente, Giovanni Lorusso, Ilaria Villanova, Michele Ficco, Matteo Gravina, Cristina Ferrari, Luca Giordano, Vincenza Granata, Dino Rubini, Nicola Maria Lucarelli, Amato Antonio Stabile Ianora, Arnaldo Scardapane

**Affiliations:** 1 Section of Radiology and Radiation Oncology, Interdisciplinary Department of Medicine, University of Bari “Aldo Moro”, 70124 Bari, Italy; 2 U.O.C. Radiologia, P.O. San Paolo, ASL Bari, 70123 Bari, Italy; 3 Radiology Unit, Department of Medical and Surgical Sciences, University of Foggia, 71122 Foggia, Italy; 4 Section of Nuclear Medicine, Interdisciplinary Department of Medicine, University of Bari “Aldo Moro”, Piazza Giulio Cesare 11, 70124 Bari, Italy; 5 U.O.C. Radiodiagnostica, Ospedaliera Vito Fazzi, 73100 Lecce, Italy; 6 Division of Radiology, Istituto Nazionale Tumori IRCCS Fondazione Pascale-IRCCS di Napoli, 80131 Naples, Italy; 7 Department of Precision Medicine, University of Campania “L. Vanvitelli”, 80138 Naples, Italy; 8 Sperimental Medicine Department, University of Salento, 73100 Lecce, Italy

**Keywords:** cardiac amyloidosis (CA), cardiovascular magnetic resonance (CMR), late gadolinium enhancement (LGE), T1 mapping, T2 mapping, extracellular volume (ECV)

## Abstract

Amyloidosis is a rare infiltrative condition resulting from the extracellular accumulation of amyloid fibrils at the cardiac level. It can be an acquired condition or due to genetic mutations. With the progression of imaging technologies, a non-invasive diagnosis was proposed. In this study, we discuss the role of CMR in cardiac amyloidosis, focusing on the two most common subtypes (AL and ATTR), waiting for evidence-based guidelines to be published.

## 1. Introduction

Amyloidosis is an uncommon heterogeneous group of diseases resulting from the presence of extracellular deposition of amyloid fibrils in the tissues. The fibrils are insoluble and derive from a variety of normally soluble precursor proteins, which misfold and assemble in an abnormal cross beta-sheet conformation.

Amyloid deposition has the potential to affect almost any organ of the body; disease is caused when the accumulation of amyloid fibrils disrupts the inherent structure and function of the affected organ.

Cardiac amyloidosis (CA) is characterized by the extracellular deposition of misfolded proteins in the myocardium, causing cellular injury and impairing compliance [1]. CA is confirmed when an endomyocardial biopsy demonstrates amyloid deposits, with the pathognomonic histological property of green birefringence when viewed under cross-polarized light after staining with Congo red, irrespective of the degree of left ventricular (LV) wall thickness. In some specific cases, diagnosis may also be confirmed if amyloid deposits within an extracardiac biopsy are accompanied by cardiac imaging.

CA results in a restrictive and/or infiltrative cardiomyopathy. Without treatment, it leads to progressive heart failure and, ultimately, death. CA is associated with the worst prognosis compared to other amyloidosis localizations [2].

Traditionally, the approach to treatment has primarily focused on providing supportive care, especially for ATTR amyloidosis. However, currently, patients with CA have access to targeted disease-modifying therapeutics; AL amyloidosis, in particular, could be treated with various combinations of chemotherapy, immunotherapy, and off-late monoclonal antibodies.

## 2. Purpose of Review

With available targeted therapeutics, a comprehensive understanding of imaging modalities becomes essential for achieving early diagnosis for patients with CA. Early diagnosis enables timely treatment and accurate monitoring of the disease progression and contributes to enhancing both the quality and length of life of patients with a historically poor prognosis disease. In this review, we discuss the role of CMR in CA, focusing on the two most common subtypes (AL and ATTR), while waiting for evidence-based guidelines to be published.

## 3. Types of Cardiac Amyloidosis

While more than 30 proteins are known to be capable of aggregating as amyloid in vivo; 9 amyloidogenic proteins mainly accumulate in the myocardium to cause significant cardiac disease. More than 98% of currently diagnosed CA results from fibrils composed of monoclonal immunoglobulin light chains (AL) or transthyretin (ATTR), a serum transport protein for thyroid hormone and retinol that is synthesized primarily by the liver; ATTR amyloidosis is further subtyped in hereditary (ATTRv) or acquired (ATTRwt or wildtipe) form.

Nevertheless, some forms (AApoAI, AApoAII, AApoAIV, Ab2M, AFib, and AGel) are very rare and CA secondary to chronic inflammatory and infectious diseases (AA), although still encountered, is now much less frequent [3]. Cardiac involvement in systemic AL amyloidosis is common (up to 75%) and, in the case of ATTR amyloidosis, it is the dominant clinical feature [4].

## 4. Prevalence of Cardiac Amyloidosis

Our knowledge of the epidemiology of CA relies mostly on real-world studies using in- or outpatient claims data or registries of diagnosed patients. These data have led to the classification of CA as a rare disorder, namely as a condition affecting fewer than 5 people in 10,000.

CA is relatively common in several settings (heart failure with preserved ejection fraction, heart failure with reduced or mildly reduced ejection fraction, conduction disorders requiring pacemaker implantation, carpal tunnel syndrome surgery, hypertrophic cardiomyopathy phenotype, or severe AS requiring valve replacement) when this condition is actively searched; that the prevalence of CA increases with age [1].

AL CA incidence is reported to be 1.6/100,000 people per year [1].

Both ATTRv and ATTRwt cardiac amyloidosis variants are frequently underrecognized but are significant contributors to diastolic heart failure; ATTRwt cardiac amyloidosis appears quite common, with recent reports placing the prevalence in as many as 10% to 16% of older patients with heart failure or with aortic stenosis [4].

## 5. Diagnosis of Cardiac Amyloidosis

The diagnosis of CA remains challenging owing to several factors, which include the relative rarity of the disease and clinical overlap with more common diseases that result in the thickening of the myocardium such as in hypertension, chronic renal failure, hypertrophic cardiomyopathy, etc.

CA is diagnosed with certainty when amyloid fibrils are found within cardiac tissue; endomyocardial biopsy and immunohistochemistry tests are still considered the diagnostic gold standard. Invasive diagnostic criteria apply to all forms of CA, whereas non-invasive criteria are accepted specifically for ATTR [4]. However, due to the invasiveness of the procedure, endomyocardial biopsy cannot be performed for all suspected patients.

Over the last year, five national or international scientific societies (ESC [1,5], DGK [6], CCS/CHFS [7,8], AHA [9,10], and JSC [11]) have published documents regarding CA, proposing novel diagnostic criteria [12,13]. The most significant innovation in the diagnosis of cardiac amyloidosis (CA), as reported by the aforementioned scientific societies, focuses on the non-invasive diagnosis of ATTR-CA.

The diagnostic approach for ATTR-CA has been substantially transformed by the ability to achieve a non-biopsy diagnosis through cardiac imaging, including echocardiography, CMR, and cardiac scintigraphy. This technique proposed by Gillmore et al. in 2016 was subsequently validated in a large cohort of CA patients and has become the cornerstone of the algorithm. The cardiac scintigraphy with bone tracers has demonstrated impressive sensitivity and specificity values of 99% and 86%, respectively, with a positive predictive value close to 100% when combined with serum immunofixation. In the latest statements, cardiac imaging is an essential part of the diagnostic algorithm.

However, even for ATTR CA, a cardiac biopsy remains necessary in the context of equivocal imaging or the co-existence of a monoclonal gammopathy.

### 5.1. Invasive Diagnostic Criteria

CA is confirmed through endomyocardial biopsy, which demonstrates amyloid deposits upon Congo red staining. Following the identification of amyloid, classification of the amyloid fibril protein is performed. Although mass spectrometry is considered the gold standard for defining the type of amyloid, immunohistochemistry or immunoelectron microscopy is routinely used for amyloid typing in specialized centers. According to ESC, DGK, and CCS/CHFS, diagnosis can also be confirmed if amyloid deposits within an extracardiac biopsy are accompanied either by characteristic features of CA by echocardiography (in the absence of an alternative cause for increased LV wall thickness) or by characteristic features on CMR (Table 1 and Table 2).

### 5.2. Non-Invasive Diagnostic Criteria

**Figure 1:**
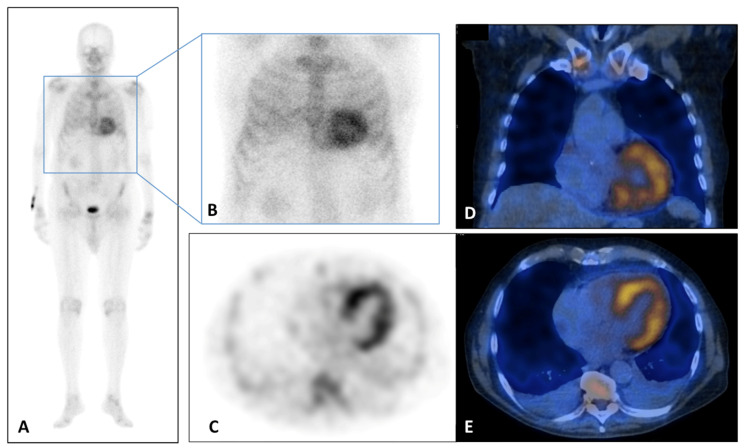
(**A**–**E**) 99Tc-Sn-HDP Scintigraphy performed in a 80 years old man with suspicious of cardiac amyloidosis and absent monoclonal protein. He presented to the emergency department for lipothymia and echocardiogram demonstrated signs of infiltrative cardiomyopathy with a LVEF of 40%, global longitudinal LV strain of −18% in absolute value. In addition, laboratory testing was significant for N-terminal pro b-type natriuretic peptide (NT-ProBNP) elevation at 5053 pg/mL (n.v. < 127.4 pg/mL). Whole Body (**A**) and static (**B**) planar bone scintigraphy, completed with tomographic imaging SPET (axial, (**C**)) and hybrid SPET/CT (coronal, (**B**) and axial, (**C**)) demonstrated moderate cardiac uptake. It correlated with score 2 of Perugini grading scale (cardiac uptake with intensity similar rib uptake), confirming the TTR amyloidosis diagnosis. LV, left ventricle; LVEF, left ventricular ejection fraction; NT-ProBNP, N-terminal pro b-type natriuretic peptide.

Cardiac ATTR amyloidosis can be diagnosed even without histological confirmation when typical echocardiographic/CMR findings (Table 2 and Table 3) are present along with scintigraphy using bone radiotracers such as 99mTc-pyrophosphate (PYP), 99mTc-3,3-diphosphono-1,2-propanodicarboxylic acid (DPD) or 99mTc-hydroxymethylene diphosphonate (HMDP) demonstrating grade 2 or 3 myocardial uptake; grade 0 stands for absence of tracer myocardial uptake and normal bone uptake, grade 1 for lower myocardial uptake than at bone level, grade 2 for similar myocardial and bone uptake and grade 3 when myocardial uptake is greater than bone). Additionally, exclusion of clonal dyscrasia is required through serum free light chain (FLC) assay, serum (SPIE), and urine (UPIE) protein electrophoresis with immunofixation (Figure 1).

Therefore, in the presence of echocardiographic/CMR findings with grade 2/3 of myocardial scintigraphy uptake in the absence of a clonal abnormality is highly specific to diagnose ATTR cardiac amyloidosis avoiding the need for endomyocardial biopsy. In cases of confirmed ATTR CA, genetic counselling with TTR gene sequencing are recommended to differentiate between ATTRwt and ATTRv forms.

## 6. Role of CMR

CMR provides highly accurate, and detailed characterization of cardiac tissue and morphology, playing a crucial role in functional assessment, providing a fundamental tool for distinguishing between CA and other hypertrophic phenocopies [14]. CMR also plays important role in the risk stratification of patients presenting with CA, which can be summarized as follows: several CMR imaging parameters have prognostic implications in patients with CA; both anatomical and tissue characterization parameters of LV are associated with outcomes in patients with CA; reduction in RV function assessed by CMR predicts mortality in patients with CA [15].

Moreover, CMR is also useful in follow-up. ESC suggests a scheme of follow-up in patients with CA, which consists of 6-month visits with electrocardiogram (ECG), complete blood tests (including N-terminal pro B-type natriuretic peptide and troponin) and echocardiography/CMR and yearly 24-h Holter ECG.

The optimal monitoring frequency of serial cardiac imaging using echocardiography or CMR is uncertain, with most reports suggesting a range between every 6–48 months, and/or in the setting of clinical deterioration [7,8].

## 7. CMR Protocol

A comprehensive CMR evaluation for CA includes morphologic and functional assessment of the left and right ventricles and atria (Table 4).

The Society of Cardiovascular Magnetic Resonance (SCMR) and the European Association of Cardiovascular Imaging (EACVI) strongly recommend a standardized protocol for image acquisition.

SCMR indicates that CMR should be performed on a minimum 1.5 T whole-body scanner; a specific surface coil with multiple coil elements (typically ≥8 elements) is highly recommended and is required to employ parallel imaging techniques that reduce scan and breath-hold times.

ECG-gating hardware and software are required and preferably incorporate vector-cardiographic gating. ECG-gating capabilities should include the ability to perform prospective gating, retrospective gating, and triggered gating techniques [16]. Updated CMR normal reference values should be used for analysis [17]. The recommended CMR protocol includes breath-hold cine balanced steady-state free procession images (b-SSFP), native and post-contrast T1 mapping with extracellular volume (ECV) measurement (T1 map acquisition is recommended in two short-axis slices and a 4-chamber view before and after contrast), and Early and Late Gadolinium Enhancement (EGE and LGE). Biventricular systolic function should be assessed by breath-hold cine b-SSFP in the short-axis stack; assessment of LGE should be performed using a phase-sensitive inversion recovery T1-weighted sequence (PSIR) on short- and long-axis images, acquired 10–15 min after peripheral bolus injection of gadolinium contrast medium [18].

## 8. Semiotics of CMR

CA morphologic and functional assessment shows typical features of restrictive cardiomyopathy: LV global wall thickening (>12 mm), predominantly at the basal segments, typically concentric in AL but can be asymmetric in ATTR CA; preserved or reduced LV systolic function (LV ejection fraction < 60%) with possible apical functional sparing in advanced case; reduced end-diastolic volume (<90 mL) with a reduced LV stroke volume index (<35 mL/m^2^); biatrial enlargement (left atrium > 41 mm, right atrium > 44 mm); atrial septum thickening (≥6 mm); and pericardial and pleural effusion (Table 5) [4,19,20].

### 8.1. T1 Mapping

T1 mapping measures the T1 signal (T1 relaxation times in ms) of each voxel in an image. T1 mapping can be calculated, either pre-contrast (native T1 value meaning longitudinal magnetization value of the myocardium before contrast) and post-contrast in order to ECV evaluation.

Native T1 values (pre-gadolinium contrast) that provide a combined signal from myocyte and extracellular space are increased in areas of amyloid deposition in both ATTR and AL patients compared with normal and HCM tissues; increased native T1 values could allow characterization and detection of the degree of infiltration [4]. In AL CA, T1 values are higher than ATTR CA [21,22]. A positive correlation between T1 values and CRM indexes of systolic and diastolic dysfunction was observed [23].

The major limitation of native T1 is the lack of reproducibility for different scanners; native T1 is easily affected by the magnetic field strength of the systems (1.5 T or 3.0 T scanners) and imaging sequences, it thereby requires the establishment of reference values for each system and imaging sequence. Despite this, according to EACVI, patients with CA could have T1 values >1050–1150 ms.

Native T1 may find utility in cases when the administration of contrast is contraindicated. A recent report demonstrated that native myocardial T1 measured by the shortened modified look-locker inversion recovery (ShMOLLI) method achieved a diagnostic sensitivity of 92% and specificity of 91% [4].

### 8.2. Extracellular Volume (ECV)

ECV measurement enables to isolate and quantify the signal from the extracellular space. To measure the ECV, a formula that exploits the T1 value before and after the contrast, corrected with the hematocrit value, is used. Current techniques require a single breath-hold and generate an ECV map automatically. The reference value for ECV is 23–28%.

Native T1 and ECV demonstrate correlations with CA severity and outcomes, creating anticipation for their application in the assessment of risk and therapeutic effects and progress monitoring and other aspects of clinical management [24]. ECV values are higher in ATTR than in AL CA, in which histology shows myocyte hypertrophy [25,26]. Unfortunately, ECV values in CA overlap with other cardiomyopathic pathologies such as acute myocardial infarction (AMI), hypertrophic cardiomyopathy (HCM), dilated cardiomyopathy (DCM), etc. [27]. Although the values of extracellular volume (ECV) are not specific, EACVI and AHA indicate that values of ECV > 40% are highly suggestive of CA. Recent studies have demonstrated that extracellular volume results elevated in the earliest phases of the disease highlight the potential role of ECV as an early disease marker [4].

### 8.3. T2 Mapping

T2 mapping measures the myocardial T2 values (T2 relaxation times [ms]) of each voxel in an image; it is a marker of myocardial edema or inflammation and a possible new aspect of the pathological evolution of the myocardium in CA [4].

Normal myocardial T2 values are reportedly 40–50 ms; however, as with T1 mapping, reference values must be established for each system.

Ridouani et al. reported that myocardial native T2 values were significantly higher in AL (63.2 ± 4.7 ms) than in ATTR (56.2 ± 3.1 ms) patients and both higher than in healthy subjects (51.1 ± 3.1 ms) [28].

Nevertheless, a substantial overlap in mean T2 values between CA and control groups has been observed [29].

In addition, myocardial T2 seems to be a significant predictor of prognosis in AL amyloidosis [11].

### 8.4. Late Gadolinium Enhancement (LGE)

The expansion of extracellular volume that results from amyloid fibril deposition within the myocardial extracellular space is accurately visualized using the administration of gadolinium-based contrast agent Gd-DTPA (gadolinium diethylenetriamine penta-acetic acid), studying the LGE. Most current gadolinium-based contrast agents are extracellular and extravascular. They do not enter intact myocytes; they accumulate within the increased extracellular space or into ruptured myocytes.

#### 8.4.1. LGE Acquisition Technique

Traditional LGE imaging techniques require an operator-determined null point, which is the inversion recovery time at which the normal myocardium appears black or “nulled”.

This can be challenging in CA: the blood pool and myocardium null together due to expansion of the extracellular myocardial volume (from amyloid infiltration); this phenomenon, known as dark blood pool, is highly sensible and moderately specific for CA.

Inversion time (TI) set to null normal myocardium. A “TI scout” cine sequence, which applies an inversion pulse at the beginning of the R-wave, can serve as a rough guide for setting the TI. However, the TI scout sequence may have slightly different readout parameters compared to the segmented LGE sequence and hence the correct TI may be up to 50 ms different between the two sequences.

Utilizing the widely available new phase-sensitive inversion recovery sequence (PSIR) for LGE, the need to optimize precise null point settings is eliminated, making LGE in CA more robust and less operator-dependent.

For clinical reporting, LGE images are typically interpreted using the 17-segment model recommended by the AHA [30].

#### 8.4.2. CMR Sequences for LGE

Pulse sequences for CMR include

- Two-dimensional segmented inversion recovery gradient echo (GRE) or b-SSFP, PSIR, or 3D sequences are preferred for patients with satisfactory breath-holding ability and adequate signal-to-noise ratio (SNR);
- Single-shot imaging (b-SSFP readout) can be performed as an optional second set or as a backup for patients with irregular heartbeat or difficulty holding their breath.

Images are generally acquired during diastolic standstill, after at least 10 min wait after contrast medium injection.

#### 8.4.3. LGE Pattern

LGE reflects both amyloid deposition in the myocardial interstitium and subendocardial ischemic changes (fibrosis) associated with microangiopathy. Although multiple LGE distributions have been described in CA, LGE has almost a pathognomonic distribution; Maceira et al. first demonstrated it is typically diffuse, subendocardial, and/or transmural on CMR [31]. LGE may be also present in the RV wall, LA wall, and atrial septum. The presence of dark blood pools, a distinctive feature observed in amyloidosis, arises from the unique kinetics of the contrast agent. In amyloidosis, gadolinium is rapidly cleared from the blood pools, leading to a contrast effect that highlights the distribution of gadolinium within interstitial amyloid depositions throughout the body [4].

**Figure 2:**
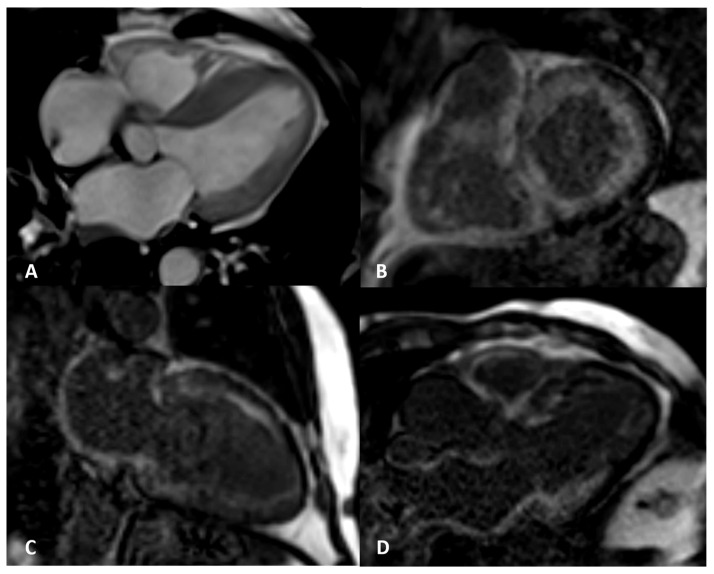
AL amyloidosis confirmed by endomyocardial biopsy. An elderly patient with NYHA III heart failure and 42% LVEF. Four-chamber balanced-steady state free precession (b-SSFP) MRI (**A**) shows symmetric thickening of the left ventricle with the septum maximally measuring 25 mm. Circumferential subendocardial LV LGE in two-chamber short (**B**) and long axes (**C**) and three-chamber b-SSFP (**D**); QALE score 12.

**Figure 3:**
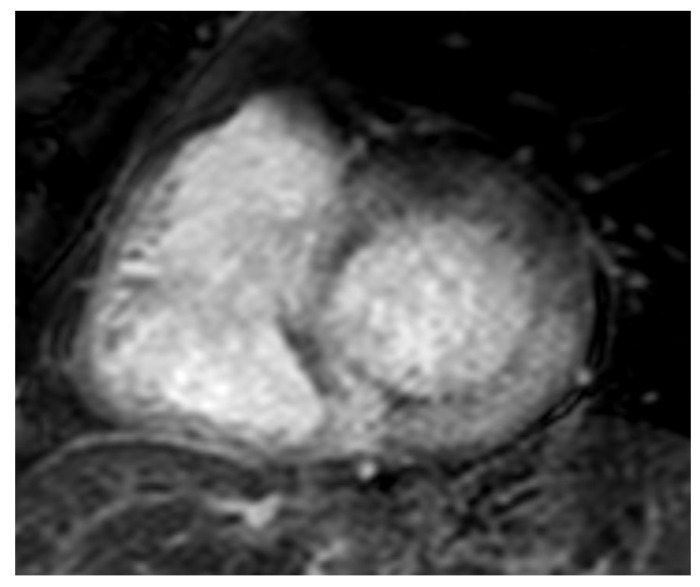
ATTR elderly patient. Transmural semicircumferential LGE pattern and symmetric thickening of the left ventricle with septum maximally measuring 21 mm.

**Figure 4:**
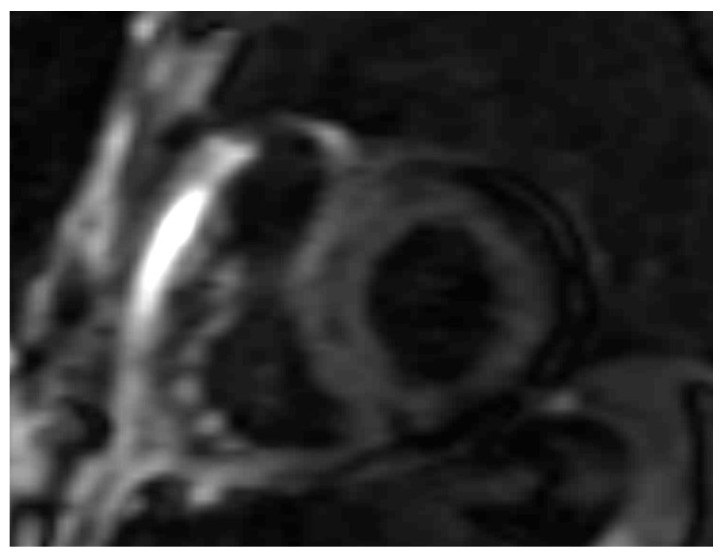
AL 68-year-old patient diagnosed with endomyocardial biopsy. T1 scout sequence shows abnormal contrast agent kinetics (Dark blood pool signal).

**Figure 5:**
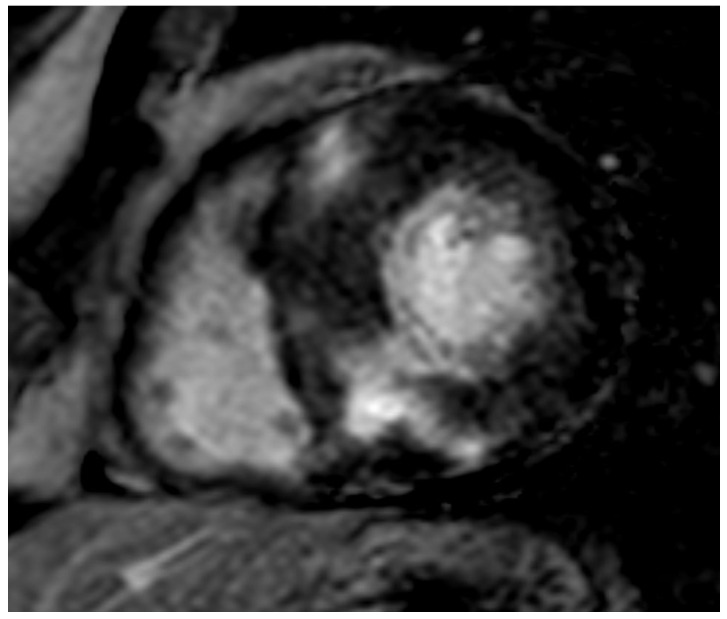
AL 64-year-old patient diagnosed with endomyocardial biopsy. PSIR sequence highlights transmural patchy LGE pattern.

**Figure 6:**
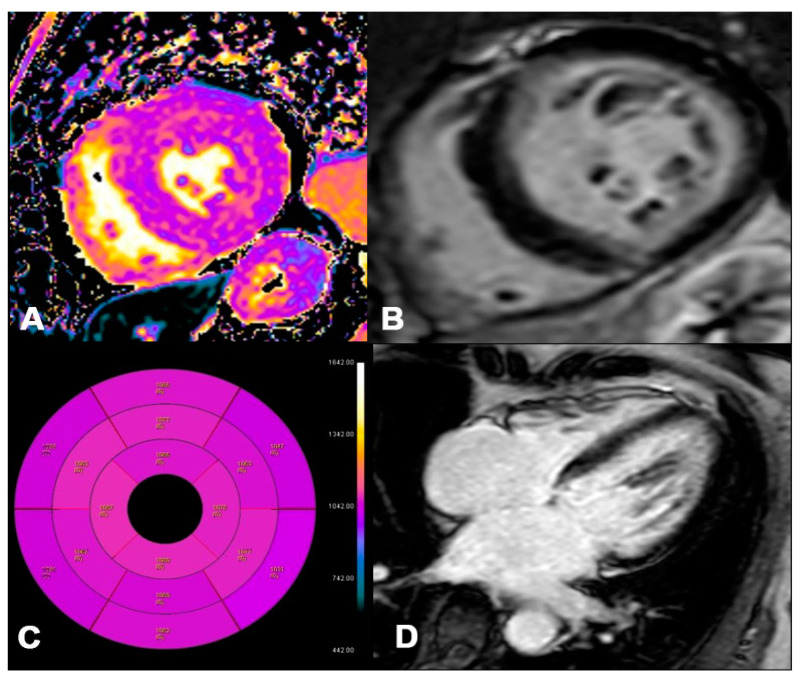
AL 73-year-old patient diagnosed with endomyocardial biopsy. T1 Native Mapping (**A**) demonstrates a slight increase in value in all entire LV walls with subendocardial patterns. Circumferential subendocardial LGE of the anterior, antero-lateral, and infero-lateral LV wall in two-chamber short (**B**) and of the SIV to the apical segment in long axes (**C**). Bullseye map of T1 Native (**D**). LV, left ventricle; LGE, late gadolinium enhancement.

**Figure 7:**
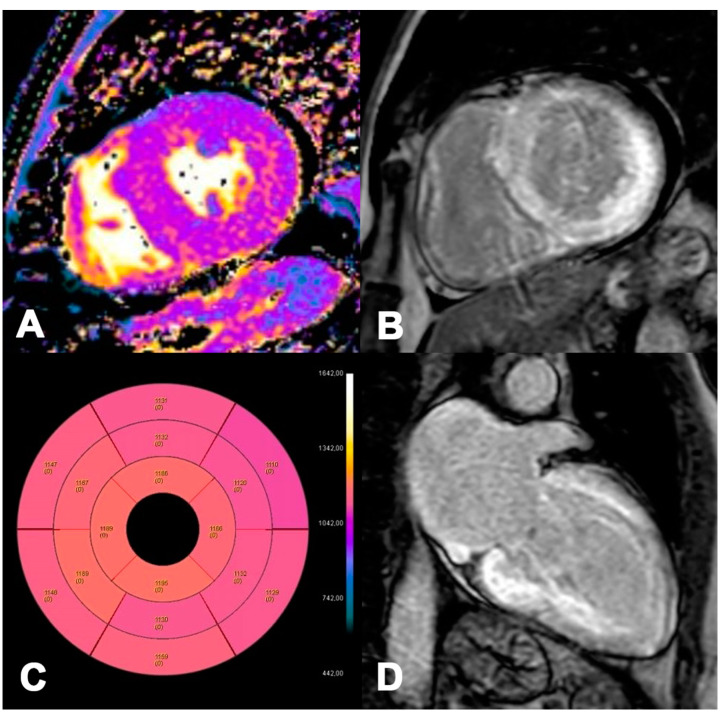
ATTR 73-year-old patient diagnosed with endomyocardial biopsy. T1 Native Mapping (**A**) demonstrates abnormally high values in all entire LV walls in cardiac amyloidosis. Diffuse transmural LV and subendocardial RV LGE in two-chamber short (**B**) and long axes (**C**). Bullseye map of T1 Native (**D**).

Subendocardial LGE is more prevalent in AL CA while transmural LGE is more prevalent in ATTR CA [32]. LGE is highly prevalent (96–100%) and more common in ATTR than AL CA but cannot distinguish between the subtypes [33] (Figure 2, Figure 3, Figure 4, Figure 5, Figure 6 and Figure 7). It has been noticed that LGE is a significant predictor of mortality both in AL and ATTR [32]. The true specificity of LGE in diagnosing CA with reference to histologic evidence cannot be accurately determined, given verification bias (typically only positive CMR cases are referred for endomyocardial biopsy). Zhao et al. meta-analysis based on seven published studies, estimated a sensitivity of 85% and a specificity of 92%, for CMR-based LGE in diagnosing CA [34].

Vermes E et al. have highlighted a base-apex LGE gradient [35], whereby LGE mostly occurs in the basal segments.

#### 8.4.4. QALE Score

**Figure 8:**
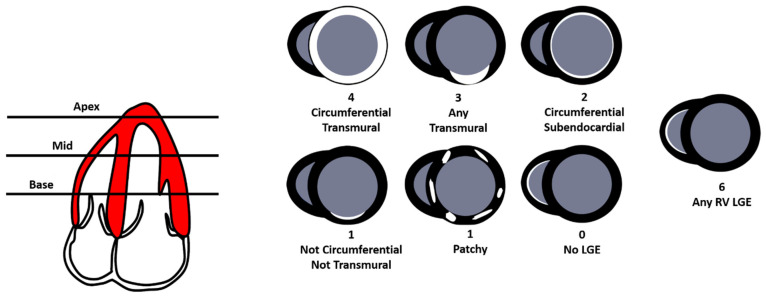
The Query Amyloid Late Enhancement (QALE) score (0–18) is performed on late gadolinium enhancement (LGE) images at the base, mid ventricle, and apex in the left ventricle (LV) and right ventricle (RV). Each LV level is scored according to the degree of LGE, with four points for circumferential and transmural LGE, three points for localized transmural LGE, two points for circumferential subendocardial LGE, one point for localized subendocardial LGE or patchy intramural LGE, and zero points for absent LGE. The maximum LV LGE score level is 12. The presence of any detectable RV LGE scores six.

Dungu, J.N. et al. [36] proposed the Query Amyloid Late Enhancement (QALE) score to identify and study the different LGE patterns in CA. The Query Amyloid Late Enhancement (QALE) score (0–18) is performed on late gadolinium enhancement (LGE) images at the base, mid ventricle, and apex in the left ventricle (LV) and right ventricle (RV). Each LV level is scored according to the degree of LGE, with four points for circumferential and transmural LGE, three points for localized transmural LGE, two points for circumferential subendocardial LGE, one point for localized subendocardial LGE or patchy intramural LGE, and zero points for absent LGE. The maximum LV LGE score level is 12. The presence of any detectable RV LGE scores six (Figure 8. The group demonstrated in a cohort of 46 patients with AL CA and 51 patients with ATTR CA that the LGE scoring system (Query Amyloid Late Enhancement) independently differentiated ATTR from AL amyloidosis (odds ratio 1.9) and, when incorporated into a logistic regression model with age and wall thickness, detected ATTR type with 87% sensitivity and 96% specificity. A QALE score ≥ 13 differentiated ATTR from the AL type with 82% sensitivity and 76% specificity. A significant positive correlation was also demonstrated between the QALE score and interventricular septal thickness, LV mass index, LV end-diastolic volume index, and LV end-systolic volume index. A negative correlation was demonstrated between the QALE score and left ventricular ejection fraction (LVEF). No significant correlation was observed between the QALE score, LV stroke volume index, and NT-proBNP [36]. Currently, a few studies have been conducted to validate the diagnostic and prognostic role of the QALE score. A study conducted by Chatzantonis G et al. on 120 patients with CA amyloidosis and 40 patients in the control group demonstrated that the QALE score significantly increases in the CA group compared to the CONTROL group [12 (7–17) vs. 4 (2–5); *p* < 0.001] [37]. Similar results were obtained by Abulizi M. et al. whereby the QALE score was significantly higher in CA than in no-CA patients (10.35 ± 5.30 vs. 3.50 ± 3.42, respectively); the optimal QALE cut-off by ROC analysis to distinguish CA from no-CA was ≥ 5 (AUC = 0.848; sensitivity 82.6%. specificity 75.0%). Contrariwise, the mean QALE scores of AL and ATTR patients did not exhibit significant differences [38]. No difference in QALE score between AL and ATTR was also found by Ridouani F. et al. in a cohort of 24 AL and 20 ATTR CA [28]. In conclusion, CMR LGE scoring seems to differentiate between amyloid types but the technique requires validation in additional prospective studies. The group of Ke Wan studied the prognostic role of QALE score; it provides a powerful independent prognostic value in AL CA; therefore, LGE ≥ 9 indicates a worse prognosis in AL amyloidosis patients with a subendocardial LGE pattern [39]. Currently, the QALE score is not routinely used, requiring further validation.

## 9. Assessment of Prognosis

Both AL and ATTR CA significantly impact the quality of life and outcome of patients, so cardiac assessment is mandatory for risk stratification and treatment decisions. Imaging plays a pivotal role in risk stratification in patients with CA.

Several CMR measures hold prognostic significance in CA, i.e., LGE presence and pattern, native T1, post-contrast T1, and multiple morphologic parameters [40]. A reduced LV ejection fraction and the presence of right ventricular LGE, RVEF, and tricuspid annular systolic excursion (TAPSE) are associated with increased mortality.

Despite the initially conflicting reports regarding LGE prognostic impact in cardiac amyloidosis, several studies have now demonstrated that the LGE pattern can indeed serve as an independent predictor of prognosis after adjustments with echocardiographic findings and blood biomarkers (NT-proBNP and troponin) [41].

The LGE pattern has prognostic implications in both AL and ATTR CA. Although LGE is valuable for prognostication in CA, its utility for quantifying myocardial infiltration is hardly limited due to variations in patterns and signal intensities. Hence, the real ability of LGE to precisely track changes over time and monitor response to treatment remains uncertain. T1 mapping has the potential to overcome these limitations [22].

Recent studies have indicated that elevated native myocardial T1 values can effectively stratify a worse prognosis in AL [26] but not in ATTR CA [42].

Alternatively, T1-derived ECV has been linked to prognosis in both AL and ATTR CA after adjusting for established independent predictors [26,33]. T2 mapping, a measure of myocardial edema, it is an independent predictor of prognosis in patients with AL CA [29].

## 10. Limitations of CMR

Although CMR is an accurate and valuable imaging modality, there are several limitations associated with its use.

CMR is expensive and may not be widely available in all healthcare facilities, leading to potential delays in obtaining imaging.

CMR examinations are time-consuming. Routine examinations, including cine sequences and late enhancement, can typically be completed within 25 min; additional time is needed for patient preparation.

Moreover, MRI requires significant patient cooperation and claustrophobic or obese patients may struggle to undergo the exam. Image quality and the duration of the examination are heavily dependent on the patient’s ability to perform repeated breath-holds of 5–10 s. Highly irregular heart rhythms can also pose challenges to image quality, often requiring the repetition of several sequences.

Furthermore, many patients undergoing CMR may have implanted cardiac devices. The primary concern with these devices is the potential for heating of the leads’ tips in MRI scanners. While efforts are being made by manufacturers to address these issues, compatibility with CMR remains a significant consideration in the development of new technologies [43].

Lastly, gadolinium-based contrast agents are commonly used in CMR for “late enhancement” imaging and myocardial perfusion assessment. However, there have been reports of nephrogenic systemic fibrosis associated with the use of these contrast agents in patients with renal failure. Consequently, contrast media should be used cautiously in patients with an estimated glomerular filtration rate (eGFR) of less than 30 mL/min [44].

## 11. Conclusions

With the aim of a less invasive diagnosis of CA that avoids myocardial biopsy, CMR plays a decisive role in the diagnosis of both ATTR and AL CA types, in particular non-invasive diagnosis of ATTR CA. The main imaging findings are based on the LGE pattern, T1 mapping, and ECV.

Research currently aims to implement the role of LGE, T1 mapping, and ECV in differentiating subtypes of CA and evaluation of prognosis.

Furthermore, new sequences such as native T1 and T2 mapping do not require the administration of contrast medium, making them particularly useful for patients with impaired renal function. With ongoing advancements in magnetic resonance technology and techniques, CMR has the potential to become pivotal for the diagnosis and follow-up of CA.

## Figures and Tables

**Table 1:** Invasive and non-invasive diagnosis of cardiac amyloidosis according to recent statements of national or international scientific societies. ATTR, transthyretin amyloidosis; CMR, cardiac magnetic resonance.

Diagnosis Criteria for Cardiac Amyloidosis			
All types		Only ATTR (with absent monoclonal protein)	
Positive cardiac biopsy	Positive extracardiac biopsy + Echocardiographic/CMR features	Grade 2 or 3 cardiac uptake at scintigraphy + Echocardiographic/CMR features	
	*		

* According to ESC, DGK and CCS/CHFS.

**Table 2:** ESC Echocardiography criteria for diagnosis of cardiac amyloidosis [3]. IVS, interventricular, septum; LV, left ventricular; LVEDD, left ventricular end-diastolic diameter; PWT, posterior wall thickness; TAPSE, tricuspid annular plane systolic excursion.

Echocardiography Criteria	
Unexplained LV Thickness (>_12 mm)	
At least 2 of: -Grade 2 or worse diastolic dysfunction -s^I^, e^I^ and a^I^ waves velocities <5 cm/s at tissue Doppler -Global longitudinal LV strain absolute value < −15%	Score ≥ 8 points: -(IVS wall thickness + PWT)/LVEDD > 0.6 3 points -Doppler E wave/e^I^ wave velocities > 11 1 point -TAPSE ≤ 19 mm 2 points -LV global longitudinal strain absolute value ≤ −13% 1 point -Systolic longitudinal strain apex to base ratio > 2.9 3 points

**Table 3:** ESC CMR criteria for diagnosis of cardiac amyloidosis [3].

CMR Criteria
Characteristic CMR findings (a and b have to be present):
Diffuse subendocardial or transmural LGE Abnormal gadolinium kinetics * ECV ≥ 0.40% (strongly supportive, but not essential/diagnostic)

CMR, cardiac magnetic resonance; LGE, late gadolinium enhancement; ECV, extracellular volume. * Abnormal gadolinium kinetics: myocardial nulling preceding or coinciding with the blood pool (Dark blood pool signal).

**Table 4:** CMR protocol for diagnosis of cardiac amyloidosis.

CMR PROTOCOL		
	Sequence technique	Note
Cine function	b-SSFP ECG gated	Short-axis, 2-chamber, 3-chamber, 4-chamber
T1 mapping pre-contrast (Native T1)	Quality controlled T1 mapping sequence	Mid and basal short-axis and apical 4-chamber views
Myocardial edema	STIR	Short-axis
Gadolinium-based non-protein bound cyclic contrast agent (0.1–0.2 mmol/kg)		
T1 mapping post-contrast (ECV estimation)	Quality controlled T1 mapping sequence	Mid and basal short-axis and apical 4-chamber Should be acquired at least 10-min postcontrast
LGE	TI scout + PSIR	Short-axis, 2-chamber, 3-chamber, 4-chamber

CMR, cardiac magnetic resonance; LGE, late gadolinium enhancement; ECV, extracellular volume; b-SSFP, balanced steady-state free precession; PSIR, phase-sensitive inversion recovery.

**Table 5:** CMR findings in cardiac amyloidosis.

CMR FINDINGS	
LV Function and Morphology	
LV function	Preserved or reduced LV systolic function
	(LV ejection fraction < 60%)
LV wall thickening	LV global wall thickening
	(>12 mm)
LV end-diastolic volume	Reduced end-diastolic volume
	(<90 mL)
LV stroke volume index	Reduced LV stroke volume index
	(<35 mL/m^2^)
Atrial size	Biatrial enlargement
	(left atrium > 41 mm, right atrium > 44 mm)
Atrial septum thickening	atrial septum thickening
	(≥6 mm)
Pericardial effusion	Pericardial effusion
Amyloid imaging	
LGE imaging	Abnormal LGE Pattern
	Diffuse LGE
	Subendocardial LGE
	Patchy LGE
	Dark blood pool signal *
Amyloid quantification	
T1 mapping pre-contrast (Native T1)	Abnormal T1 mapping. Native T1 values are increased in areas of amyloid infiltration
T1 mapping post-contrast (ECV estimation)	Values of ECV > 40% are highly suggestive of CA.

CMR, cardiac magnetic resonance; LV, left ventricular; LGE, late gadolinium enhancement; ECV, extracellular volume. * Dark blood pool signal: myocardial nulling coinciding with the blood pool.
